# Impact of the single site order in LTC: exacerbation of an overburdened system

**DOI:** 10.1186/s12913-023-09670-7

**Published:** 2023-06-20

**Authors:** Farinaz Havaei, Joanie Sims-Gould, Sabina Staempfli, Thea Franke, Minjeong Park, Andy Ma, Megan Kaulius

**Affiliations:** 1 School of Nursing, University of British Columbia, T201-2211 Wesbrook Mall, Vancouver, BC V6T2B5 Canada; 2 Center for Hip Health and Mobility, University of British Columbia, 2635 Laurel Street, Vancouver, BC V5Z 1M9 Canada; 3 Simon Fraser University, 8888 University Dr, Burnaby, BC V5A 1S6 Canada

**Keywords:** Single site policy, Impact, COVID-19, Nursing human resources, Mixed-methods

## Abstract

**Background:**

The long-term care (LTC) sector has been at the epicentre of COVID-19 in Canada. This study aimed to understand the impact that the Single Site Order (SSO) had on staff and leadership in four LTC homes in the Lower Mainland of British Columbia, Canada.

**Methods:**

A mixed method study was conducted by analyzing administrative staffing data. Overtime, turnover, and job vacancy data were extracted and analyzed from four quarters before (April 2019 – March 2020) and four quarters during the pandemic (April 2020 – March 2021) using scatterplots and two-part linear trendlines across total direct care nursing staff and by designation (i.e., registered nurses (RNs), licenced practical nurses (LPNs) and care aids (CAs)). Virtual interviews were conducted with a purposive sample of leadership (10) and staff (18) from each of the four partner care homes (*n* = 28). Transcripts were analyzed in NVivo 12 using thematic analysis.

**Results:**

Quantitative data indicated that the total overtime rate increased from before to during the pandemic, with RNs demonstrating the steepest rate increase. Additionally, while rates of voluntary turnover showed an upward trend before the pandemic for all direct care nursing staff, the rate for LPNs and, most drastically, for RNs was higher during the pandemic, while this rate decreased for CAs. Qualitative analysis identified two main themes and sub-themes: (1) overtime (loss of staff, mental health, and sick leave) and (2) staff turnover (the need to train new staff, and gender/race) as the most notable impacts associated with the SSO.

**Conclusions:**

The results of this study indicate that the outcomes due to COVID-19 and the SSO are not equal across nursing designations, with the RN shortage in the LTC sector highly evident. Quantitative and qualitative data underscore the substantial impact the pandemic and associated policies have on the LTC sector, namely, that staff are over-worked and care homes are understaffed.

**Supplementary Information:**

The online version contains supplementary material available at 10.1186/s12913-023-09670-7.

## Background

The long-term care (LTC) sector has been the epicenter of COVID-19. In Canada, among all COVID-19 cases during the first wave of the pandemic, one in five belonged to LTC residents and one in 10 to LTC workers [1]. In this timeframe, LTC residents accounted for 81% of all COVID-19 deaths in the country with some jurisdictional variations (e.g., no cases in New Brunswick vs. 97% of all deaths in Nova Scotia). While Canada’s overall COVID-19 mortality rates were considered lower than rates in most countries in the world, LTC rates were shockingly high— double the rate of other member countries of the Organization for Economic Co-operation and Development (OECD) [2]. In response to the concerning circumstances in LTC and to ensure the health and safety of staff, residents and their families, the Public Health Agency of Canada (PHAC) mandated several rapid redesign and resource redeployment practices such as strict visitation restrictions, COVID-19 screening, use of personal protective equipment (PPE) and the “one high risk site” staffing policy. Emerging pandemic evidence shows despite important contributions to slowing down the spread of infection, these policies were complex and had some unintended consequences for LTC homes, their users, and care providers [3, 4].

### Context

Examining the nuances of the “one high risk” policy (also known as the Single Site Order (SSO)) and its implementation in LTC requires a broad understanding of the sector’s unique context. LTC homes, also known as nursing homes, continuing care facilities, and residential care homes, provide a wide range of health and personal care services for Canadians with medical or physical needs requiring access to 24-hour nursing care, personal care and other therapeutic and support services [5]. Across Canada, 2,076 LTC homes deliver services to 198,220 Canadians majority aged 65 or older, representing 3% of Canada’s total older adult population [6]. These care homes are either publicly (46%) or privately (54%) owned. Privately owned homes may have a private for profit (29%), private not-for-profit (23%) or fully private (2%) structure [5]. An overwhelming majority of LTC residents live with one or more impairments (physical or cognitive) and therefore have highly complex care needs that are met by nearly 254,000 LTC workers nationally [7].

LTC workers are relatively evenly distributed across fulltime (39.3%) and parttime (38.9%) employment with a smaller proportion (~ 18%) holding casual employment [7]. A minute proportion of LTC staff are contracted from employment agencies (~ 4%). Despite these national trends, variations exist across jurisdictions and individual care homes. More consistent, however, is the distribution of LTC workers’ classifications and personal demographics. An overwhelming majority (~ 90%) of LTC workers are direct care providers, including unregulated support workers (also known as care aides [CAs]; 65.5%) followed by regulated nurses (registered nurses [RNs] and licensed or registered practical nurses [LPNs]), physicians, and allied health providers (26.1%). Indirect care workers make up a small proportion of LTC staff (~ 10%) [7]. LTC workers are adversely impacted at the intersection of age, gender, and race; racialized middle-aged or older immigrant women are over-represented in LTC and earn less than their counterparts working in acute care settings [8, 9]. These workers also have higher rates of self-rated ‘poor’ physical health, primarily due to the heavy physical nature of LTC work [10].

The SSO was first implemented on March 26th, 2020, in British Columbia LTC homes, and three weeks later in other Canadian provinces including Ontario and Alberta [11, 12]. The SSO prevented LTC staff from employment at more than one ‘high risk’ site, defined as LTC, assisted living, or provincial mental health facilities [13]. Other worksites such as acute care settings were not considered ‘high risk’ and therefore were excluded from this definition. The SSO was not equally applied to all LTC workers. While some worker classifications (e.g., nurses, support workers) were included in the SSO, others were excluded (e.g., nurse practitioners, pharmacists, physicians, and dieticians) from the policy [14].

In British Columbia, the implementation of the SSO involved care homes’ acquiring their staff’s actual and preferred place/s of employment information for submission to their representative health authority. To determine the staff cohort for each LTC home, health authorities reviewed their information along with staffing requirements and available staff resources for each home. Decisions were informed mostly based on operational factors such as safe staffing and continuity of care; when feasible, staff’s personal circumstances and preferences were considered [13].

To uphold continuity of care, the SSO recommended that regular fulltime employees who also worked as regular parttime or casual employees at any other ‘high risk’ site to be assigned only to their regular fulltime worksite, resulting in a greater loss of casual and parttime employees for LTC homes [13]. The SSO also recommended that regular parttime and casual employees who worked at more than one worksite to rank their worksite preference. Consequently, the SSO resulted in a smaller pool of staff at individual care homes as well as decreased work hours and increased wage losses for LTC workers [12]. To compensate for the loss of staff and work hours, care homes encouraged all employees to maximize their hours regardless of their employment status. Regular parttime and casual employees were encouraged to work regularly scheduled fulltime hours, and regular fulltime employees were requested to work overtime [13].

Preliminary pandemic evidence has suggested that the SSO (and other pandemic-related factors) exacerbated long-standing staffing shortages in LTC homes. For example, a survey study of nearly 4000 support workers from 94 randomly selected and stratified LTC homes in Western Canada found one quarter of respondents worked in more than one LTC home and 15% worked in non LTC sites before the pandemic [15]. These support workers, on average, worked for 16 h a week at a home other than the primary LTC home where they held a regular position [15]. Although it has been suggested that employers exploit casual staffing as a strategy for financial savings, staff identified a variety of beneficial reasons for working for multiple employers including greater flexibility in scheduling and increases in pay [8]. A case study of LTC workers in British Columbia found the SSO compromised staff mental health and quality of care mainly due to exacerbating LTC staffing shortages [3]. Consistent with this case study, a national survey of over 5000 LTC homes found that 77% reported an increase in the number of overtime hours, 71% reported an increase in absenteeism and 85% reported other staffing challenges such as critical shortages among direct care employees (e.g., nurses and support workers) during the pandemic [16]. Critical staffing shortages are defined as a shortage of staff, particularly in key roles (such as directors of care, nurses, or support workers) that had an impact on the quality of resident care and employee safety [16].

While the available literature has identified valuable information regarding the impact of the pandemic and related policies (such as the SSO) on staff and resident outcomes, most research in this area has been based on leaders’ and staff’s perceptions and reports. To our knowledge, these survey findings have not yet been triangulated with other data sources such as administrative data. As such, the aim of this study was to evaluate the impact of the SSO on LTC homes and their staff utilizing a mixed-methods study involving the analysis of routinely collected administrative data and in-depth interviews with leadership and staff.

## Methods

Data collection took place between February and April 2021 in partnership with four publicly funded LTC homes across three municipalities (Vancouver, Richmond, Surrey) and two health authorities (Vancouver Coastal [VCH] and Fraser Health [FHA]) in British Columbia, Canada. Table 1 provides an overview of the partner care home characteristics. Quantitative data were used to track potential changes in LTC homes’ staffing practices before versus during the implementation of the SSO, and qualitative data focused on gaining a nuanced understanding of the policy impact from the perspective of LTC leadership and staff.

**Table 1:** Characteristics of participating LTC + facilities

	LTC #1	LTC #2	LTC #3	LTC #4
	**Seaside**	**Lake Bay**	**The Manor**	**Rosewood**
Municipality	Mission	Vancouver	Richmond	Vancouver
# staff	~ 250	~ 400	~ 280	~ 160
# residents	~ 151	~ 250	~ 250	~ 130

To ensure an integrated knowledge translation (iKT) approach, a steering committee of 10–15 LTC stakeholders was established. The steering committee was composed of leadership, nursing, support workers and resident and family representatives from the four partner care homes and/or their respective health authority and met regularly to inform the direction of the research and to offer consultation on all aspects of the project. Select members of the steering committee formed a data subcommittee to guide the quantitative component of the project including data identification, operationalization, and extraction. Harmonized ethics approval was obtained from the Research Ethics Boards of the University of British Columbia, the partner care homes and/or their respective health authority [H20-03967].

### Quantitative methods

The quantitative component of the study entailed extracting and analyzing retrospective and prospective staffing data from the administrative database of each partner care home or from that of their representative health authority. Aggregated staffing data were extracted at quarterly intervals between April 2019 and March 2021. The period between April 2019 and March 2020 (four quarters) was defined as ‘pre-COVID-19’, and the following period between April 2020 and March 2021 (four quarters) as ‘during COVID-19’. The research team and the data subcommittee worked together to identify staffing indicators (a) most important in relation to the SSO and (b) consistently operationalized across the partner care homes.

#### Quantitative measures

The most pertinent staffing indicators in relation to the SSO were identified as staff overtime, turnover (voluntary and involuntary), vacancy, sick time, and leave of absence. Among these indicators, overtime, turnover, and vacancy rates were consistently operationalized across at least three of the four partner care homes and were subsequently included in the analysis. Sick time and leave of absence were excluded from this analysis due to inconsistent operationalization across each partner care home.

Table 2 provides care homes’ operational definitions of the staffing indicators. Given that direct care support workers (i.e., CAs) and nurses (i.e., RNs and LPNs) make up the greatest proportion of LTC staff [7], staffing indicators focused only on these designations and were aggregated across each of the three designations.

**Table 2:** Operational definitions

Administrative staffing data	Definition	Consistently operationalized
**Overtime rate** (in percentage)	The number of productive hours worked by all direct care nursing employees that were callback or non-callback overtime hours, expressed as a percentage. (Note that productive hours include all hours defined as productive by Finance and worked by regular, temporary, full or part-time employees.)	LTC 1, 2, 3, 4
**Turnover rate** (in percentage)		LTC 1, 2, 4
Voluntary turnover rate	The number of direct care nursing employees leaving voluntarily (resignations, retirements) over the total number of employees, expressed as a percentage.	
Involuntary turnover rate	The number of direct care nursing employees leaving involuntarily (layoffs, disciplinary, administrative) over the total number of employees, expressed as a percentage.	LTC 1, 2, 4
**Vacancy rate** (in percentage)	The number of vacant regular positions for direct care nursing employees over the total number of regular positions, in percentage.	LTC 1, 2, 4

#### Quantitative data analysis

Staffing data were analyzed using descriptive statistics and data visualization methods whereby separate scatterplots per care home for each indicator were generated, descriptively illustrating the change across eight quarters between April 2019 and March 2021. Scatterplots including data from a minimum of three of the four care homes were also created. Each of these plots were supplemented with a two-part linear trendline presenting the overall trends pre- and during pandemic, as well as smoothed data curves for each care home. The two-part linear trendlines were created by separately fitting simple ordinary least-squares regression models to the pre-pandemic datapoints, and the during-pandemic datapoints. Trend slopes were also annotated to the plots to facilitate comparison of trend changes pre and post pandemic onset. Trends were obtained across total direct care nursing staff and specifically by direct care designation (i.e., RN, LPN, CA). Inferential statistics were not used due to the aggregated nature of each datapoint and limited sample size (i.e., only eight time points for each home).

### Qualitative methods

#### Recruitment and sampling

The research team worked with four partner LTC homes to recruit leadership team members and staff for interviews. Purposive sampling [17] was used to focus on maximizing diversity across job titles and across LTC homes, while selecting individuals that were especially knowledgeable about the SSO. Leadership team members had to be English speaking and staff had to be English or Cantonese speaking.

Leadership team members circulated an email to their staff that invited them to participate in the study. Staff members then contacted the research team to schedule a date and time for their interview and were sent a consent form to read, sign, scan and return to the researchers via email. Participants were not provided with an incentive to partake in the interviews, and interviews took place during staff working hours.

A total of 10 leadership team members and 18 staff participated in the qualitative interviews (*n* = 28).

Leadership team participants held job titles including CEO, Executive Director, Nurse Manager, CA Manager, Director of Human Resources, and Clinical Operations Supervisor. Out of the 10 leadership team participants, eight identified as female. Staff job titles included RNs, CAs, Laundry Aides, Chefs, and Housekeepers. Out of the 18 staff, one identified as male; 80% were over the age of 40; and length of time working at the facility ranged from 6 months to 37 years. Each care home had at least two staff and two leaders participate in an interview.

#### Data collection

After obtaining informed consent from participants, interviews were conducted virtually via Zoom at a time most convenient for the participant. Only the interviewees, the interviewer and a note taker were present at the time of the interview. Interviews ranged in length from 45 to 60 min.

#### Measures

Interview guides were internally developed by the research team with a focus on evaluating the impact that the SSO had on LTC homes and their staff (see Table 3 in the Appendix for example interview questions).

#### Qualitative data analysis

We applied a thematic analysis, where data were sifted, charted and sorted in accordance with key issues and themes using five steps: (1) familiarize; (2) generate initial codes; (3) search for themes; (4) review themes; and (5) define and name themes [18]. The steps are discussed briefly below. Each interview was fully transcribed verbatim. Initially TF read through all the interview field notes and a couple of the transcripts (familiarize). Through team meetings [SS, TF, JSG] a preliminary thematic framework was developed to simplify and focus on specific characteristics of the data (e.g., overtime, staff turnover). The thematic framework consisted of main themes based on key issues and commonalities emerging from the field notes and initially reviewed transcripts (generate initial codes). Using NVivo 12 software, two team members [SS and TF] coded a subset of interviews based on the thematic framework, with discussion among team members, as new codes and sub-themes were identified (search for themes). Full paragraphs were coded so that contextual meaning was not lost. Data were then summarized by charting illustrative quotes that best exemplified the themes (review themes). As part of the interpretive process a series of team meetings were held to discuss the data for common themes and sub-themes (define and name themes).

## Results

### Quantitative

#### Overtime

**Fig. 1:**
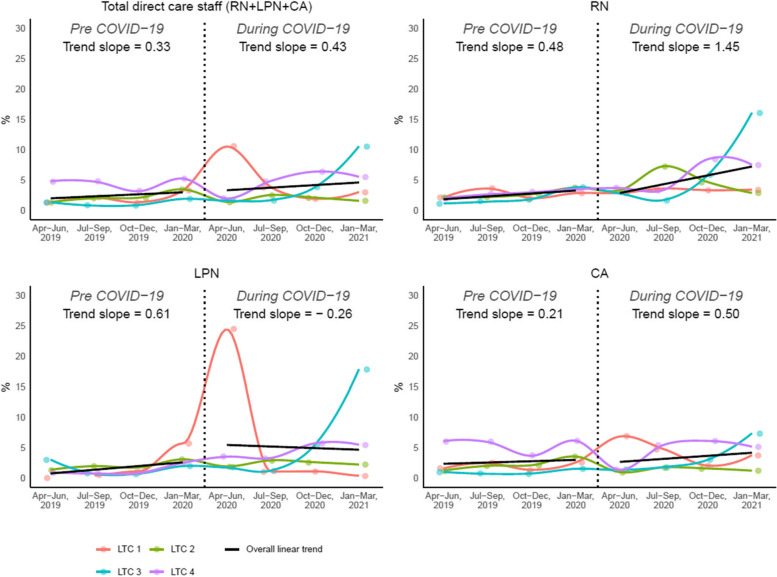
Overall and individual trends for overtime rate

Figure 1 demonstrates the overall and individual care home trends for the rate of overtime. The overall trend showed increases in total direct care staff’s rate of overtime throughout, with a faster rate of increase for during-pandemic quarters compared to pre-pandemic. Pre-pandemic, the overtime rate increased by 0.33% for every quarter whereas during COVID-19, the overtime rate increased by 0.43% every quarter. Cross-classification analyses showed that the rate of increase steepened during COVID-19 for RNs (pre-pandemic: +0.48% per quarter, during: +1.45% per quarter) and CAs (pre-pandemic: +0.21% per quarter, during: +0.50% per quarter), but declined for LPNs (pre-pandemic: +0.61%, during: -0.26%).

#### Turnover (voluntary and involuntary)

**Fig. 2:**
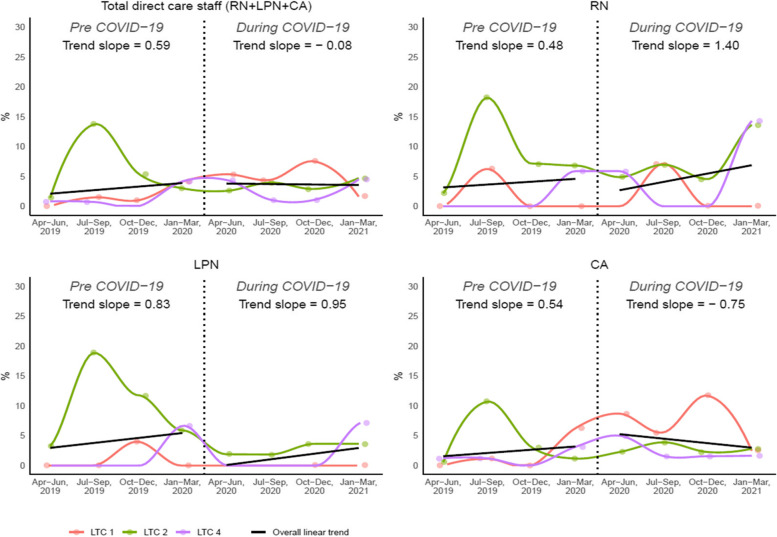
Overall and individual trends for voluntary turnover rate (LTC 1, 2, 4)

**Fig. 3:**
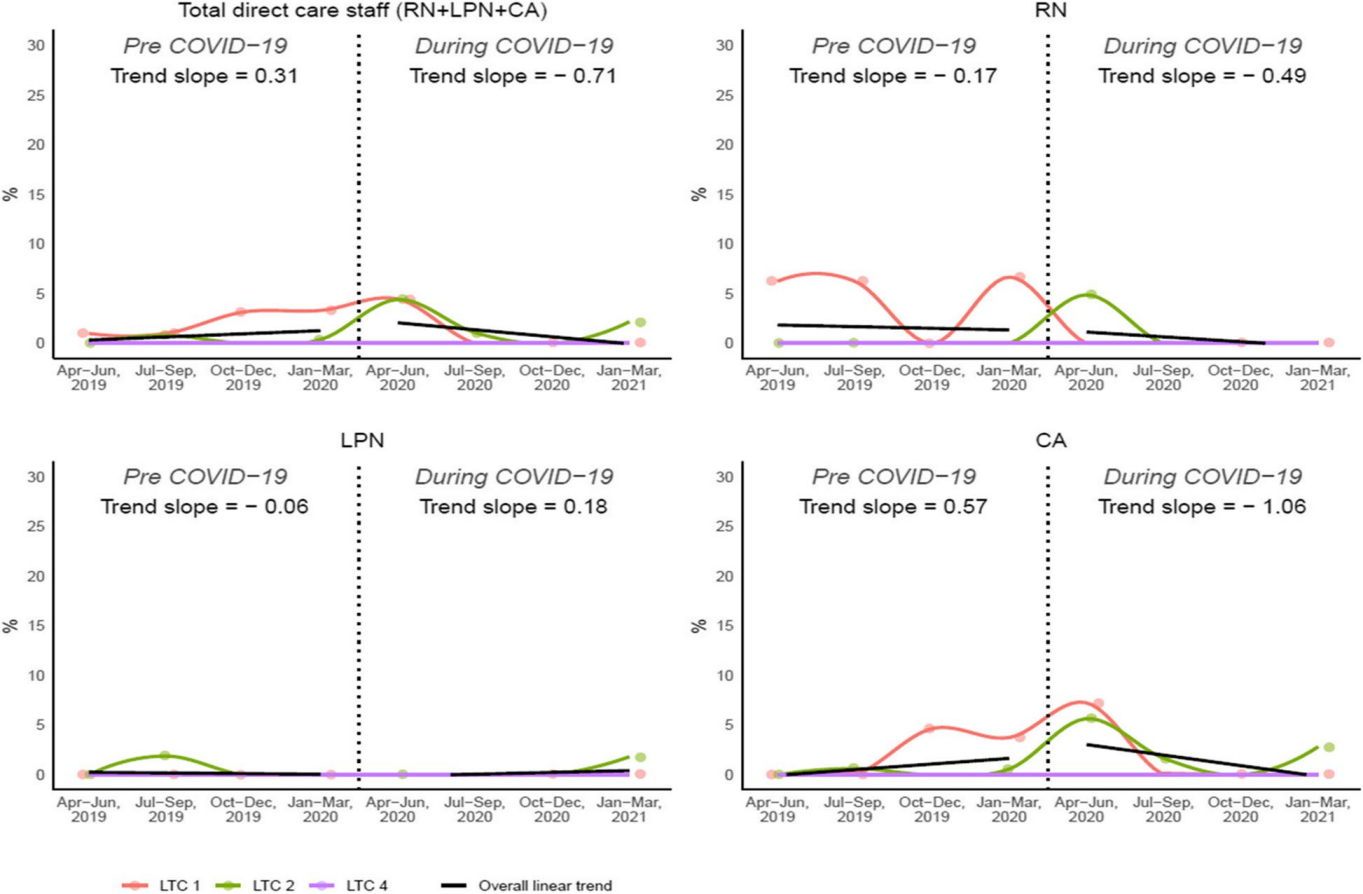
Overall and individual trends for involuntary turnover rate (LTC 1, 2, 4)

Figures 2 and 3 demonstrate the overall and individual care home trends for the rate of voluntary and involuntary turnover respectively. The overall pre-pandemic voluntary turnover rate for all direct care staff increased faster than during the pandemic. The pre-pandemic rate increased by 0.59% every quarter but remained relatively stable during the pandemic (decreasing by only 0.08% per quarter). For RNs and LPNs, the voluntary turnover rate increased faster during the pandemic (RNs: +1.40% per quarter, LPNs: +0.95% per quarter) than pre-pandemic (RNs: +0.48% per quarter, LPNs: +0.83% per quarter). For CAs, however, the rate increased pre-pandemic but decreased during the pandemic (pre-pandemic: +0.54%, during: -0.75%).

The overall involuntary turnover rate for all direct care staff demonstrated an increasing trend across the pre-pandemic quarters, with an average increase of 0.31% per quarter. During the pandemic, however, the involuntary turnover rate demonstrated a decreasing trend over time (-0.71% per quarter). During the pandemic, the involuntary turnover rate of CAs showed the greatest rate of decrease across the nursing designations (-1.06% every quarter). For RNs, the rate decreased by 0.49% per quarter during the pandemic, while for LPNs, the involuntary turnover rate increased by 0.18% per quarter.

#### Vacancy rate

**Fig. 4:**
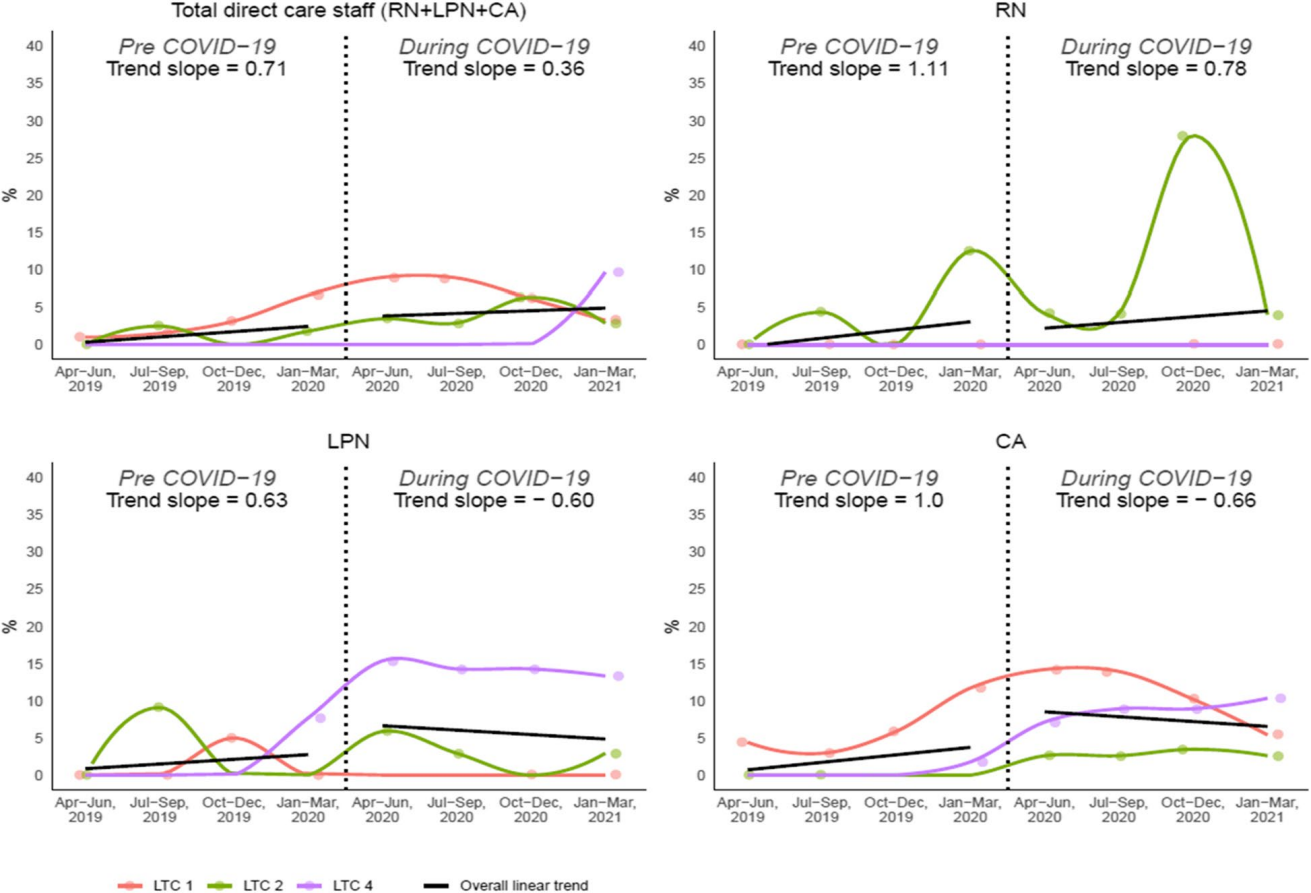
Overall and individual trends for vacancy rate (LTC 1, 2, 4)

Figure 4 demonstrates the overall and individual care home trends for the rate of vacancy. The overall vacancy rate for all direct care staff increased across the pre-pandemic and during-pandemic quarters. The rate of increase was higher pre-pandemic than during the pandemic (pre-pandemic: +0.71%, during: +0.36%). The overall pattern of change was reflected by the trends for RNs, with the RN vacancy rate increasing by 1.11% pre-pandemic, but increasing more slowly (0.78%) during the pandemic. For LPNs and CAs, the trend in vacancy rates changed from increasing pre-pandemic (LPNs: +0.63% per quarter, CAs: +1% per quarter) to decreasing during the pandemic (LPNs: − 0.6% per quarter, CAs: − 0.66% per quarter).

### Qualitative

Qualitative analysis identified two main themes and sub-themes: (1) overtime (loss of staff, mental health, and sick leave) and (2) staff turnover (the need to train new staff, and gender/race) as the most notable impacts associated with the SSO.

#### Overtime

##### Loss of staff

The SSO resulted in loss of staff particularly from the casual pools. As an example, one LTC home’s casual pool dropped from 24 available staff members to nine. To compensate for the smaller staffing pool, LTC homes relied on overtime to meet resident needs. Staff were frequently scheduled to work overtime and worked multiple consecutive shifts, with many staff members scheduled to work night shifts and again the following morning.

Suddenly at both my sites our casual pools disappeared, and so we were struggling to fill shifts and so suddenly you have a ton of overtime because there’s no casuals to pick up shifts that are vacant. – [Leadership, The Manor]

For people who are working over one FTE [full time equivalent], even though I guess, they were doing that before and I worry about burnout, I worry about people who work nights and then, you know, we’re scheduling them in the daytime to make up those hours. The toll on people. However, having said that, I know some of these folks, as I said, were working you know huge amount of hours kind of regularly so I’m not trying to be paternalistic if that was their thing then I’m glad that we’re able to support them in this way I don’t think it’s the thing to do, you know forever. – [Leadership, Lake Bay]

LTC homes that required bilingual staff due to their resident population experienced heightened staffing challenges. Given that these homes faced difficulties filling adequate fulltime staff roles and heavily relied on casual workers to meet staffing needs pre-pandemic, the implementation of the SSO (and subsequent loss of casual pools) resulted in prominent staffing shortages in these homes and the need for overtime.

I think for Rosewood additional layer of challenges that the language requirement … if we go back to the question that you asked me earlier how confident, I am in sustaining this single site I think single site itself is fine, but I’m not confident to sustain a bilingual staff, you know staff population under single site. – [Leadership, Rosewood]

Leadership and staff emphasized the extreme shortage of registered and licenced practical nurses in the LTC sector. Interviewees suggested that seniors’ care has been greatly devalued and finding nurses even before the pandemic was quite challenging. It was noted that one of the facilities had not received a single RN application in the entire past year, and another facility was unable to recruit RNs or LPNs despite posting for open roles for multiple months.

RNs are like unicorns in long term care. RNs do not exist; it was really hard before the pandemic to recruit RNs for LTC. – [Leadership, The Manor]

##### Mental health

Concerns about overtime and working consecutive shifts throughout the week led to a perceived reduction in psychological and physical health of staff.

Because most of my regular staff are taking overtime… they are the one who’s mostly filling up the gaps in between and I saw how big of an impact this is in terms of stress, burnout, you know and mental wellness among the staff, but we had no choice we just had to carry on, this is our commitment. – [Leadership, Seaside]

Exhaustion due to loss of staff, increased workloads and not taking sick leave when needed played a key role in the increased musculoskeletal injuries observed in support workers (i.e., CAs). After long shifts, staff were often susceptible to injuries.

Yeah and another aspect about being short on staff and the same, I really, really appreciate the care aides, so they did an amazing job they are basically they move in casement they work 16 hours. … so they did all their parts, you know, and not only physical, but now I can see there’s a price for that, because they overall work their body and last week one in our team one care aide sustained a muscular skeletal injury and it was it could be preventable, but I believe because she’s a very responsible care aide. But because her body’s too tired she had suffered this injury, so this is another aspect, and we can say this is a tax we are being right now. [Staff, Seaside]

The pandemic itself brought on a lot of anxiety and stress for LTC staff, particularly around inadequate PPE provision before vaccinations were made available in LTC. Interviewees feared contracting the virus and spreading it to loved ones. Staff emphasized that the pressure of the last year was not due to the homes they worked in, but due to the nature of the pandemic and the subsequent policies and restrictions that were implemented with the intention of keeping residents and staff safe (e.g., strict infection prevention and control (IPAC) measures and visitation restrictions).

Out there were lots of apprehension with the staff. All of us were kind of scared to come to work because there was, I think swabbing was not done right away. And then day by day the progress it’s just in place but apprehension and anxiety of not knowing your enemy of work it’s invisible enemy. – [Staff, Lake Bay]

Obviously, they were all broken-hearted and they were all scared even I remember, I was scared that what we take home with us, you know we all have kids and family, and we were all thinking the same way, because we didn’t have a vaccine, then, and things like that, and we were always. As much as they told us to be careful and we had to change masks and do donning doffing every room, we have to change every mop in each room, we have to change the gloves and everything, but we still have this level of thinking, our heart either worried that you know what if we catch something and take it on with us. - [Staff, The Manor]

Along with the loss of staff due to the SSO, the loss of volunteers and family members was highlighted as a major contributor to the reduction of quality of care that residents received, and the additional workload staff had to take on. Family and volunteers often provide the ‘daily living’ pieces of care such as helping residents with personal grooming (e.g., cutting hair, brushing teeth), eating food, and engaging in exercise. Both staff and leadership felt that family and volunteers should be considered as essential as CAs and companions. Staff had to continuously witness the isolation many residents experienced, which interviewees noted caused trauma and hardships for many staff.

Housekeeping staff felt a lot of stress during outbreak times, seeing residents isolated to rooms was distressing. – [Leadership, The Manor]

She used to visit her mother every day after work okay. She served her mother with her dinner and then also get her mother to brush teeth and then put her mother to bed before she leaves at night, and on the weekends, she goes in the morning, so make sure that her mother gets to brush your teeth okay after the lockdown the first dentist appointment her mother got eight cavities. Because the reason behind that was always COVID-19 … she was really upset she said she used to help her mother with her teeth brushing every day, at least once a day she gets to brush your teeth…, but after the lockdown nobody helped her. Because the reasons they have they’re so busy they just don’t have time to help her mother for brushing her teeth. – [Staff, Lake Bay]

##### Sick leave

To compensate with the mental and physical health implications of working under strenuous conditions, staff called in sick. However, their strong sense of duty to the LTC home and the residents led to staff feeling apprehensive about calling in sick. Even when staff felt symptoms or illness, they felt immense guilt if they were to call in sick as this was perceived to be letting other staff down by imposing their responsibility on others.

It’s not uncommon for people to work 7 or 8 days in a row, then feel tired and call in sick. – [Staff, Lake Bay]

I’ve never asked for sick leave, it’s seeming like those who asked for sick leave it’s not much more than other places. But they really are too tired, like they work for sometimes more than 6 days. When they’re so tired, the quality of work will not be very good. So, this is all a problem. Because a lot of people do 7 days, some people to 8 days, that is that is, in a row people work 7 days or 8 days. They’d be tired afterwards. – [Staff, Rosewood]

#### Staff turnover

##### The need to train new staff

Following the loss of staff due to the SSO, LTCs hired and trained new staff who were often inexperienced and unfamiliar with facility operations. The process of training new staff was taxing for original staff who were already exhausted with their own work responsibilities. For example, kitchen staff felt as though their job description changed; they worked at the care homes for the elders and were now sequestered in the basement with no contact with the elders. Many reported finding this extremely difficult and contributing to their sense of job dissatisfaction.

There were some hirings done, and then the problem was we got a lot of staff that never worked in the kitchen before so that was more of a challenge for some of the regulars to pick up and help them right. They [leadership], had to hire staff that didn’t work other place so that left a lot of people coming in that didn’t have healthcare experience right, so I think it’s been a hard year for us in the kitchen, I feel like it’s been a lot of extra work for the regular staff. Staff challenges and adaption to the work environment, it was tough, but a lot of time it took to educate the newcomers to get on board it’s not like ABC to get them who are not experienced in that field. They had to understand the routine get along and get going right still there is ups and downs and challenges, but we all work as a team to make sure, things are going, the best of our knowledge right yeah [Staff, Seaside]

##### Gender and race

The staff demographic in LTC homes are predominantly racialized females, with English as a second language [8, 9], who are at an increased risk of exposure to workplace discrimination. Further to this, a lack of personnel and the need to train new staff created a greater burden for current employees and a continued deepening of racial and gender inequalities as the overburdened staff were primarily racialized immigrant women. There was increased anxiety for staff when residents were unable to fully communicate with staff because of language barriers, particularly regarding the use of PPE and increased IPAC measures. Interviewees noted that residents were less likely to express their needs when interacting with staff who were of a different ethnocultural background.

It’s a sector where there’s a lot of predominantly women and it’s also predominantly immigrant women in a lot of the positions, especially the care aide positions. You know English isn’t usually their first language. - [Leadership, The Manor]

He said ‘You [N-word], go back to where you come from.’ But, he wasn’t demented or something, the patient was more alert, but he was very upset […] it’s not right, but he was really upset. When I tr[ied] to de-escalate the problem, he was very upset and that [was] what he said. - [Staff, Lake Bay]

## Discussion

This study has several key findings. Overall, the results from this study indicate that the SSO exacerbated long-standing staffing-related challenges in LTC. The overall rates of overtime, turnover (voluntary and involuntary), and vacancy trended upward before the pandemic, illustrating the staffing crisis in this sector prior to the onset of COVID-19. The qualitative and quantitative data demonstrate that the pandemic and subsequent SSO and related policies resulted in even greater staffing challenges for LTC homes.

The rate of overtime increased more steeply during the pandemic than before. This phenomenon was described in detail by participants in the qualitative interviews, with staff and leadership linking the increase in overtime to the loss of casual staff as a direct result of the implementation of the SSO. Interviewees revealed that increased overtime may have been an indirect product of LTC- and pandemic-specific factors (including the SSO): LTC workers that experienced a deterioration of their mental and physical health needed time away from work to recover, furthering LTC staffing shortages that were resolved through more overtime. This finding is consistent with a recent study that found that pandemic management policies such as the SSO and strict visitation compounded LTC staffing shortages and heavy workloads, which in turn increased mental health symptoms and absenteeism and vice versa [3]. In this context, and due to the dire shortage of LTC providers nationally [16], overtime was the only viable solution available to LTC leaders to ensure adequate staffing to meet basic resident needs.

The increase in rate of overtime was not equal across direct care designations: the overtime rate increased at a speedier pace for RNs and CAs, with RN overtime increasing most drastically. This might be because of a more prominent shortage of RNs in the LTC sector and, therefore, an over-utilization of existing RNs to meet staffing needs. Pre-pandemic evidence from the United States used payroll-based staffing data to show half of LTC homes in the country met staffing standards 20% of the time at most, with RNs driving these staffing shortages [19]. The increasing pattern of overtime is noteworthy and of particular concern: an extensive body of evidence that links nurse overtime to increased burnout, job dissatisfaction, turnover, and patient safety incidents [20, 21]. Of note is that the declining overtime trend for LPNs was surprising and may be explained by a markedly different staffing pattern that occurred in one of the four care homes as shown in Fig. 1. Related to this finding, while we observed that RNs demonstrated opposite patterns of vacancy rate (increasing) than LPNs and CAs (decreasing) during the pandemic, vacancy rates declined for all three classifications during the pandemic compared to pre-pandemic (in the case of RNs, the vacancy rate increased at a slower rate during the pandemic). This finding makes sense in the context of increasing overtime that was used to cover nurse vacancies. Furthermore, RNs’ pattern of vacancy rate is consistent with the extreme shortage of RNs in LTC.

The quantitative data also pointed to differing outcomes for the three direct care classifications in other staffing data. To illustrate, the decrease in rate of voluntary turnover during the pandemic for CAs was opposite to the increasing pattern observed for regulated nurses during the same time period. This pattern may have been a result of job dissatisfaction and unequal employment opportunities. Job dissatisfaction is one of the most important contributing factors to nurse turnover, [22] and while the evidence suggests that all three nursing classifications experienced increasingly negative feelings about their job due to working under more precarious conditions (e.g., increased staffing shortages, overtime, workplace discrimination), they did not have the same employment opportunities available to them. RNs and LPNs had a greater number of job prospects during the pandemic (e.g., public health, vaccination clinics, etc.) available to them compared to CAs, enabling these regulated nurses to more easily act on any feelings of job dissatisfaction [23]. On the contrary, CAs had little choice but to continue working under increasingly severe working conditions.

This finding may also suggest that pandemic-specific factors interacted to further the existing race and gender inequalities most prominently for already disadvantaged LTC employees. Research from the United States found CAs as the most disadvantaged group of LTC workers with respect to education, availability of health insurance, and family and financial resources [24]. In Canada, one in five women workers are racialized and employed in the lowest paying and most precarious of caring jobs that carry a high risk of exposure to COVID infection and less likely to have access to important protections such as paid sick leave or health benefits; one-third of all women workers work in high-risk jobs and the large majority are employed as CAs [25].

Finally, involuntary turnover showed a decreasing trend for CAs and RNs but a slightly increasing trend for LPNs during the pandemic. This staffing indicator reflects employees involuntarily leaving the organization due to layoffs, disciplinary or administrative reasons and therefore is a less meaningful staffing indicator than voluntary turnover to evaluate in the context of the SSO.

Qualitative data added additional context for the patterns we observed in the administrative staffing data: an overall upward slope for overtime and vacancy rate had a detrimental effect on remaining staff. It was explained that employees worked more (through an increase in overtime, working back-to-back shifts, and not taking sick time when necessary) which negatively impacted employee mental health. This finding is consistent with research evidence linking nurse overtime to poor mental health [21].

Training new staff was highlighted as a particular stressor for original employees that were already overworked before the additional training duties were included in their roles. Feelings of work overload and not taking sick time when needed were discussed as factors that lead to increased musculoskeletal injuries for staff, and a potential factor for decreasing quality of care for patients. These accounts are consistent with previous research that identified the sick time policy during the pandemic as inflexible and as the most inappropriate aspect of the LTC work environments according to LTC workers’ reports. Previous research has also found a link between increased workload and negative nurse and patient outcomes [26, 27].

Consistent with previous research [26], the interviews painted a picture of a work environment fraught with stress and exposure to traumatic events. Staff and leadership described a perception of lack of safety in their workplaces, particularly with not having adequate protection against contracting COVID-19 (i.e., PPE, vaccines). Further, the prevention of visitation from family and volunteers meant both an increase in workload for LTC workers to ensure that residents were adequately cared for and exposing staff to seeing residents in isolation and psychological distress. Finally, data from the interviews suggested that staff were exposed to racism and discrimination within their workplace from residents. The experience of racism in any workplace is immensely harmful but could be even more so in the LTC sector when considered with the fact that LTC workers are often individuals with more limited access to financial and social supports [24]. Additionally, fewer employment opportunities and reduced job mobility, as elucidated above, could mean that certain LTC workers (i.e., CAs) could be in a position of experiencing a deeply harmful work environment without the ability to find employment in a safer situation.

Taken together, the qualitative and quantitative data point to immense challenges experienced by LTC leadership and staff during the pandemic. It is clear that the SSO interacted with many other ongoing challenges that the LTC sector faced be findings (e.g., negatively impacted mental health, reduced perceived safety) reflect the scope of implications this pandemic has brought onto the LTC sector.

### Suggestions

Through the completion of this study, it also became clear that consistently operationalized administrative data across LTC homes is sorely lacking. The inability to include numerous key staffing indicators (such as sick leave, leave of absence, etc.) hampered our ability to garner a robust understanding that the SSO and pandemic had on staff and their resident care provision. An investment in consistently captured, defined and publicly available data in this sector is an important and necessary step in garnering a better understanding of how to improve outcomes for LTC residents and staff.

Finally, the results of this study point strongly to the need to address the systemic issues that have plagued the LTC sector, and most specifically, the need to bolster the nursing shortage within LTC. Very few staffing policies could have been implemented without issue in the LTC sector given the long-standing lack of investment in this area and staffing challenges. Effective resident centered care delivery is highly dependent upon adequate and appropriate types of care providers. Thus, to improve resident care, it is imperative that the working conditions in LTC are improved to boost recruitment and retention of nurses, particularly RNs, to this sector. Without addressing the shortage, LTCs will suffer from the same disastrous outcomes when the next health crisis occurs.

### Limitations

We acknowledge the challenge we encountered in evaluating the isolated impact of the SSO in the context of a crisis that triggered many changes in the LTC sector, including concurrent adoption of several new policies. The design of this study did not allow for an isolated evaluation of only the SSO, but rather, provided a more global overview of the effect that the onset of the pandemic and implementation of subsequent policies within the first year of the pandemic had on the four participating LTC homes.

Additionally, as described above, the availability of quantitative data proved a limitation in this project. The variability in definitions for nursing staffing data has been well established in the literature [22] and was a challenge in our study as well. The inconsistent operationalization of quantitative data limited our ability to compare and contrast all staffing indicators across all partner care homes. Further, the data that were available may not have fully represented staff behaviour and outcomes. To illustrate, vacancy rates may not have captured employees taking leave of absences or receiving re-assignments (i.e., their role may have appeared to remain filled even if staff were not actively on staff). Similarly, the voluntary turnover rate would not have captured if a staff member was still technically an LTC employee but not taking any shifts. The infrequent timepoints of the data, partly due to their quarterly nature, were another limitation, hindering our ability to determine the statistical significance of the noted trends. Finally, the findings of this study should be cautiously generalized to other LTC homes, contexts, jurisdictions, and time periods as it is unclear if staff experiences in the partner care homes during this specific period in time (i.e., the beginning of the COVID-19 pandemic) are representative outside of our specific study population and data collection period.

## Conclusion

The results of this mixed-methods study indicate that the pandemic and subsequent SSO contributed to major challenges for LTC leadership and staff in a sector that was already experiencing notable difficulties. The implementation of the SSO resulted in the loss of casual staff, which lead to an increase in overtime (for RNs and CAs), voluntary turnover (for RNs and LPNs), and vacancy (for RNs). In particular, the shortage of RNs was particularly evident in the administrative data and noted as highly impactful through interviews with LTC workers. Leadership and staff explained that working above and beyond regular hours and taking on additional job duties (such as training new staff), in addition to general stress of working on the frontlines of a pandemic, lead to exhaustion and a reduction in mental health. Interviewees also revealed troubling work environments, such as the experience of racism in a sector largely comprised of socially and economically disadvantaged women, as a result of the pandemic and SSO. Government investments are warranted to aid the LTC sector in improving working conditions that will foster leadership and staff’s capacity to deliver quality and safe resident centered care.

### Supplementary Information

**Additional file 1.**

## Data Availability

Data are available upon reasonable request and approval from the Research Ethics Boards of the University of British Columbia, the partner care homes, and/or their respective health authority.

## Appendix

**Table 3:** Example interview questions

**Leadership interview questions** 1. How do you feel about the SSO being used in your setting? 2. What kind of information or evidence are you aware of that shows whether the SSO works in your setting? 3. What challenges has the SSO presented? 4. What costs were incurred to implement the SSO?
**Staff interview questions** 1. When you received the public health orders restricting workers to one high risk site only how did your organization respond? 2. What are some challenges the SSO presented? 3. What is your experience with the SSO? 4. With respect to COVID-19 what do you think are the biggest issues? Do these problems affect men and women differently? If yes, how?

## Abbreviations

CA: Care Aide
FHA: Fraser Health Authority
iKT: Integrated Knowledge Translation
IPAC: Infection Prevention and Control
LPN: Licensed Practical Nurse
LTC: Long Term Care
PPE: Personal Protective Equipment
RN: Registered Nurse
SSO: Single Site Order
VCH: Vancouver Coastal Health
