# Supplementary material for: Impact of the single site order in LTC: exacerbation of an overburdened system
Source: BMC Health Serv Res. 2023 Jun 20;23:666. doi: 10.1186/s12913-023-09670-7 (PMC10283221; doi:10.1186/s12913-023-09670-7)
Supplement: Supplementary file 1 — Additional file 1. [file 12913_2023_9670_MOESM1_ESM.docx]

Supplement 1. Vignette: Housekeeping

My name is Lorena, a 34-year-old female housekeeper, working at a nursing home. I have worked with my facility for two years. My family, husband and two kids, immigrated to Canada seven years ago from the Philippines. I work with mostly females, and many are Filipino which is nice to be able to speak Tagalog with my colleagues. That being said, the majority of residents are white, and we do experience a fair share of racism in the workplace. My first experience with blatant racism was when I noticed a commotion between a resident and her roommate. I attempted to deescalate the situation, and the resident called me the N word and said to go back to where I came from.

This happened on one of my first days. I remember it so clearly because I asked my colleague whether this is something I should bring up with our supervisor. My co-worker chuckled and said that this is just part of the job, they just don’t include it in the job description, and I should get used to it. Over time, I think I became de-sensitized to these remarks. But they still hurt, I just am quicker to move on after hearing or experiencing racist comments and incidences.

Understaffing was always the case in most all long-term care facilities, the pandemic simply worsened an existing crisis. During the pandemic, because of hygiene measures and low staffing (due to staff sickness, symptoms, or burnout), I felt as though I was constantly running from room to room. Many days at work felt like endless bedsheets to change, floors to mop, and surfaces to disinfect. It is quite exhausting work especially when my colleague calls in sick last minute because they don’t feel well. When this happened, I felt like I couldn’t leave my shift because if I hadn’t cleaned this residents’ room, they would not have a clean bed for the night.

There are so many things which bring me happiness through my work. I love seeing residents smile and so I try to frequently crack jokes with them and their family. With the amount of work though, it’s become increasingly difficult to carve out time to chat with residents and their family between cleaning rooms. I felt terrible for the residents and family members when visitation was highly restricted and window visits were the only option. I saw that window visits helped the residents, but it only benefitted those few who had windows in their rooms near the ground floor. For those without windows or who resided above level three, it was extremely lonely and isolating for them. Many of the residents have not been outside of the facility, nor have they seen or touched their families in over a year. But despite all these challenges, many residents still carry positive attitudes and are so kind and understanding towards staff. Something that really stuck to me is one of the residents who saw that I was having a rough day and said “Lorena, you’re such an angel, keep your peckers up”. As staff, we try our best to keep the residents happy. It brings me joy when I see the residents being entertained by the rec staff.

I hope that my story can be one voice among many who may be too busy or tired to speak. I hope that after the crisis the pandemic brings, we can keep our peckers up and draw more attention to supporting staff who provide the services for those needing care.
